# Spatiotemporal variation in fish species distribution and abundance in the Vaishav stream, Kashmir Himalaya–India

**DOI:** 10.1371/journal.pone.0316280

**Published:** 2025-02-18

**Authors:** Gowhar Rashid, Rahul Singh, Abhinav Kumar, Prabhu Paramasivam

**Affiliations:** 1 Department of Zoology, School of Bioengineering and Biosciences, Lovely Professional University, Phagwara, Punjab, India; 2 Department of Nuclear and Renewable Energy, Ural Federal University Named after the First President of Russia Boris Yeltsin, Ekaterinburg, Russia; 3 Department of Technical Sciences, Western Caspian University, Baku, Azerbaijan; 4 Refrigeration &Air-condition Department, Technical Engineering College, The Islamic University, Najaf, Iraq; 5 Department of Mechanical Engineering, Karpagam Academy of Higher Education, Coimbatore, India; 6 Chitkara University Institute of Engineering and Technology, Centre for Research Impact & Outcome, Chitkara University, Rajpura, Punjab, India; 7 Department of Mechanical Engineering, Mattu University, Mettu, Ethiopia; Sher-e-Kashmir University of Agricultural Sciences and Technology of Kashmir, INDIA

## Abstract

Exploring the intricate dynamics of aquatic ecosystems present study investigates the spatio-temporal variations in the ecological parameters of the fish community within the Vaishav stream, Kashmir Himalayas. Monthly field investigations were conducted at three distinct sites (SI, SII & SIII) throughout the four seasons (winter, spring, summer, autumn) from November 2019 to October 2020. The findings encompass a total of 630 specimens belonging to 11 fish species, three orders *Cypriniformes*, *Siluriforms* and *Salmoniformes* and four families including *Cyprinidae*, *Nemachelidae*, *Siluridae* and *Salmonidae* were reported from the study sites. Among collected specimens, *Cypriniformes* were dominant with nine species followed by order *Siluriformes* and *Salmoniformes* with one species each. Out of eleven fish species, six fish species belongs to family *Cyprinidae*, three to *Nemachelidae*, one to *Siluridae* and *Salmonidae* each. The analysis, employing non-metric multidimensional scaling (NMDS), Principal component analysis (PCA), Analysis of similarity (ANOSIM) and Per-mutational multivariate analysis of variance (PERMANOVA) on fish abundance data highlighted significant differences among the various sites but not across seasons. The results unveil a diverse occurrence and distribution pattern of fishes from upstream to downstream. Furthermore, diversity metrics confirm higher diversity index values downstream, indicating a more conducive environment for fish survival. Jaccard’s index reveals greater similarity in fish fauna between site-II and site-III than site-I and site-III in terms of overlap of fish species composition. The study concludes that anthropogenic activities in the stream catchment area have led to a reduction in fish diversity and abundance, with landscape features significantly influencing fish abundance in this unique Himalayan ecosystem.

## 1. Introduction

Fishes are considered as the best biological indicators for perceiving any alteration in water chemistry caused by various anthropogenic activities. Nowadays, the management of fish diversity and the related ecosystems seems to be a major concern, as many fish species are disappearing due to improper and delayed conservative measures. Fishes have a high degree of variation due to geographic dispersion, alteration of streams and the surroundings, stream fragmentation, natural force and presence or absence of nonnative species [1]. Natural variations among fish population are due to difference in altitude, physical habitat, water quality, water temperature and environmental variables [2]. The various aspects of fish development hold significant importance in ichthyology. Examining the composition, abundance, and distribution of juvenile fish species provides essential data, especially for commercially valuable species. Recent findings highlight a concerning trend, revealing that only 14% of the world’s rivers and fish populations remain unaffected by human-induced degradation, while a striking 86% have been adversely impacted. Additionally, freshwater bodies are declining at a rate twice than observed in marine and estuarine environments [3]. The IUCN’s listed 80 species of freshwater fishes as extinct in its Red List of threatened species, of which 16 species are mentioned in the year (IUCN, 2020) [4]. The possible reasons of extinction of fish species seems to be due to pollution, discharging of wastewater, habitat loss, overfishing, defective dams, draining wetlands, introducing invasive species, wildlife crime, and climatic change [5]. Generally fish fauna shows extreme sensitivity to quantitative and qualitative changes in aquatic environment thus are considered as are one of the most vulnerable taxonomic groups.

India stands as one of the world’s biodiversity hotspots, ranking tenth globally with a count of 2,500 fish species, including 930 freshwater and 1570 marine species [6]. Fish meat, among animal dietary resources, plays a pivotal role as a primary source of animal protein consumed globally, contributing significantly to income generation, particularly in developing countries [7]. The Kashmir valley, situated in the Himalayan mountain system of the sub-tropical oriental region, is renowned for its intricate network of lentic and lotic ecosystems harboring distinctive fish species [8]. The River Jehlum, a lifeline and the lone river of Kashmir, has been extensively explored by ichthyologists, revealing a diverse indigenous fish community, including Schizothorax spp., *Glyptothorax* spp., *Triplophysa* spp., *Barbus* spp., *Labeobarbus* spp., *Corbitis* spp., *Silurus* spp., *Crossochilus* spp., *Nemachilus* spp., and exotic trout species such as *Oncorhynchus mykiss* and *Salmo trutta fario* [9]. Notably, *Schizothorax* species dominate the fish fauna of the Kashmir valley, exhibiting uniqueness compared to the broader Indian fish fauna [10]. Unfortunately, certain *Schizothorax* species, including *Schizothorax richardsoni*, have witnessed a near-vanishing act from the water bodies of Kashmir [11]. The fishery resources of the River Jhelum are facing a decline due to various anthropogenic activities in recent years [12]. Assessing anthropogenic impacts on the region and formulating effective conservation strategies necessitates precise information on stream fish communities. Seasonal climate fluctuations significantly influence fish community dynamics, particularly in subtropical water bodies, where temperature variations and river flows play a pivotal role [13]. While numerous studies have explored fish biodiversity across India, research on Himalayan hill stream fish communities remains limited. Understanding the arrangement of fish assemblages in lotic environments is crucial for population ecology and aids in biodiversity conservation efforts. This study is aimed at if the fish community features (alpha diversity) respond to the environmental factors along the longitudinal gradient. In addition, we assume that the fish composition varies similarly among the sites but has a different composition. Lastly, we propose that the environmental factors are crucial in influencing the fish diversity and composition.

## 2. Materials and methods

### 2.1 Study site description

**Fig 1:**
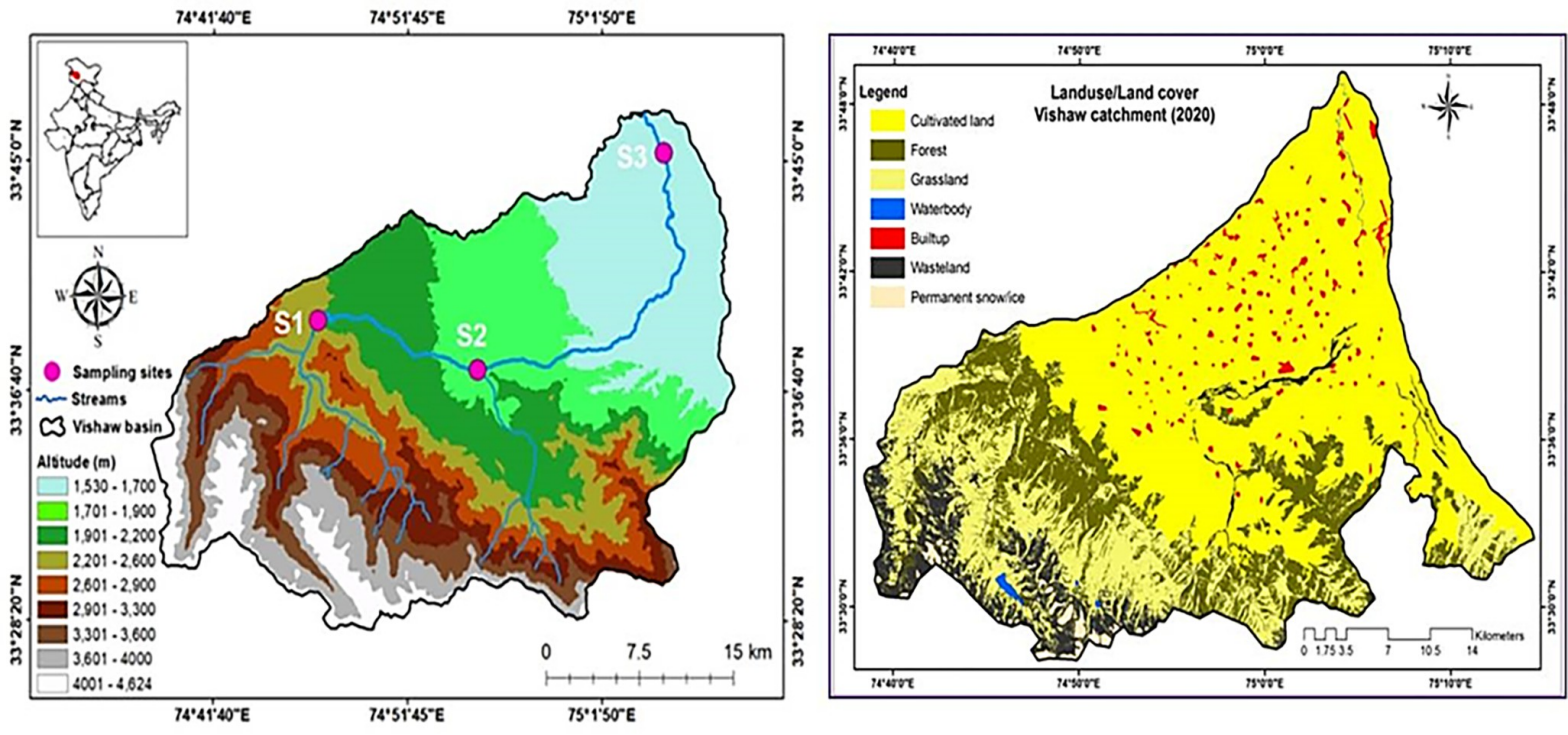
Map showing the course and location of selected sampling sites. Source: Generated by Survey of India (SOI) topographic sheets (Arc-GIS 9.0) software Landsat ETM Landsat 8 OLI satellite data, 2020.

The current study was carried out in Vaishav stream on the north-western slopes of the Himalayan Mountain range (Pir Panjal) of Kashmir, India. Vaishav stream originates from the Kousarnag, lake which is located in Pir Panjal range of district Kulgam having coordinates of 33°39 to 33°65ʹʹ N latitude and 74°35" to 75°11ʹʹ E longitudes (Fig 1, Table 1). It drifts in the form of waterfall in Aharbal then flows mainly through two districts namely Kulgam and Anantnag [14]. The study region experiences a moderate environment with cold, wet winters and warm summers. Climatic conditions are widely categorized into four seasons as spring (March-May), summer (June-August), autumn (September-November) and winter (December-February). To minimize any long-term effects on fish assemblages and after doing a comprehensive site survey for suitability, site selection criteria was used which is a crucial standard for fishing operations [15]. Site -I (Watoo, Reshinagar) is a head water site lies between geographical coordinates of 33°39"19 N latitude and 74°47"9 E longitudes at an altitude of 2187 masl. Site -II (Kulgam) is located 32 km downstream of Aharbal, a middle stream reach site draining a sedimentary calcareous watershed (limestone and shale), lies between geographical coordinates of 33°37" 55 N latitude and 74°53" 13 E longitude at an altitude of 1969 masl. Site -III (Arwani) is the last station chosen for the study is located 51 km downstream of Aharbal, at an altitude of 1724 masl within geographical coordinates of 33°37" 26 N latitude and 74°55" 25 E longitude. It is located in urbanized area with settlements in the vicinity. The base map for stream course and location of three different selected sites along with land use/cover was prepared from the Survey of India (SOI) topographic sheets, i.e., 43 K/10, 43 K/12, 43 K/14, 43 K/15, 43 O/1, 43 O/2 and 43 O/3 on 1:50,000 scale with the help of Arc-GIS 9.0 software assigning UTM, WGS 1984, 43N zone projection system. Land use/ land cover map of the study area 2020 were generated from Landsat 8 OLI satellite data, 2020 respectively.

**Table 1:** Geomorphological attributes of different sampling stations.

Attributes	Site-I	Site-II	Site-III
**Altitude** (masl)	2266	1882	1534
**Stream segment**	Upstream	Midstream	Downstream
**Position**	Latitude 33°39"19 N Longitude 74°47"08 E	Latitude 33°37"26 N Longitude 74°55"25 "E	Latitude 33°45"24 N Longitude 75°024"E
**Habitat type**	Riffle	Riffle & pool	Pool
**Substrate**	Sand, gravel, stones, silt & clay	Pebbles, cobbles, boulders	Sand, silt & clay
**Riparian vegetation**	Acacia, Apple Orchards Popular, Salix, pinus & Grass	Acacia, Apple, Orchards Popular,Salix & Grass,	Acacia,Popular, Salix & Grass,
**Land use**	Forests & Horticulture Wasteland,	Agricultural, Horticulture& urban Settlements	Agriculture & Urban Settlement

### 2.2 Fish sampling

Sampling was conducted monthly from November 2019 to October 2020, strategically avoiding diurnal variations and each site was surveyed once at the end of each month and no specific permits were required for field site access or sampling in this study area as the study was conducted in [publicly accessible lands, non-protected areas, or describe applicable designation]. The work followed all relevant regional and national regulations governing research activities in natural water bodies. Fish sampling was executed by a local fisherman from the study area utilizing a cast net equipped with knot-to-knot heavy sinkers to ensure rapid settling at the bottom. Utilizing the indigenous cast netting technique [16], In this study, fish sampling was conducted using a traditional cast net expertly handled by a local fisherman. The cast net used had a diameter of 1.2 meters and a mesh size of 1.3 to 3.0 centimeters [17], which is well-suited for capturing small to medium-sized fish species in the Himalayan streams of Kashmir Valley. This traditional approach aligns with the local fishing practices and minimizes environmental disturbance, ensuring representative sampling of the local fish diversity. Monthly sampling sessions, involving multiple trials within a 500-meter stretch [18,19].

### 2.2a Ethical approval

All applicable international, national, and/or institutional guidelines for the care and use of animals were followed during the present study. All the protocols used in the present study have been approved by Animal Ethical Committee registered under R.No. 801/Go/RE/S/2003/CPCSEA.

### 2.3 Water sampling procedure

The water samples were collected from three different sampling sites from November 2019 to October 2020 on monthly basis (Table 1). The sample preservation, transportation, and analysis were completed within 24 hours using the recommended procedures [20]. Temperature, pH, total dissolved solids, conductivity, and dissolved oxygen were analysed at sample locations. While as turbidity, free carbon dioxide, total alkalinity, total hardness, calcium hardness, magnesium hardness, nitrate, sulphate and total phosphorus were measured in laboratory by using standard titrimetric procedure adopted by [20].

### 2.4 Statistical data analysis

Methods and Equipment to collect results is listed in Table 2. In this study, the fish community pattern was systematically investigated across three sampling sites and four seasons, employing multivariate statistical analysis. The exploration of relationships between various environmental factors and fish community composition on tempo-spatial patterns was a central focus during the experimental period. To create an ordination based on similarity or dissimilarity among sites and seasons, the non-metric multidimensional scaling (NMDS) ordination technique was employed [21]. The "vegan" package facilitated the calculation of pairwise dissimilarity between sites using Bray-Curtis dissimilarity [22]. Data suitability for factor analysis (FA) was confirmed through Kaiser-Meyer-Olkin (KMO) and Bartlett’s sphericity tests, both of which indicated the dataset’s appropriateness for Principal Component Analysis (PCA) at p<0.05 [23]. PCA, applied to reorganize the datasets into a new set of variables, helped reduce dimensionality. The orthogonal principal components (PCs) were then presented in decreasing order of significance [24]. Standardization of water quality data via Z-scale transformation and normalization were undertaken to address classification errors stemming from variations in data dimensionality [25]. The PCA analysis was executed using the "prcomp" function and "ggbiplot" package in the R programming language. Subsequently, per-mutational multivariate analysis of variance (PERMANOVA) was conducted to investigate paired differences in fish community composition with sites and seasons, utilizing the Adonis pair function of the R package [26] with a statistical significance of 9999 permutations. Alpha diversity metrics, detailed in Table 3, were employed to estimate community structure, and a T-test was used to determine significance among three sites and four seasons based on 11 fish species and the number of individuals [27].

**Table 2:** Methods and equipment to collect results.

Parameters	Unit	Method	Equipment
**Air Temperature**	°C	Manual	Graduated Mercury °C Thermometer
**Water Temperature**	°C	Manual	Graduated Mercury °C Thermometer
**Turbidity**	NTU	Electronic Turbidity meter	Naphalo Turbity meter
**pH**	-	Digital pH meter	HANNA HI98129
**Electrical Conductivity**	μs/cm	Digital conductivity meter	Labtronics LT-17
**Dissolved Oxygen**	mg/l	Digital DO meter	Lutron type DO-5510
**TDS**	mg/l	Digital TDS meter	Tayser-T3
**Total Alkalinity**	mg/l	Titrimetric Method (APHA-2012)	Phenolphthalein indicator (Titration with 0.01N HCl)
**Free CO**_**2**_	mg/l	Titrimetric Method (APHA-2012)	Phenolphthalein indicator (Titration with NaOH)
**Total Hardness**	mg/l	Complexometric titrimetric Method (APHA-2012)	0.01 N EDTA using Eriochrome black T
**Calcium Hardness**	mg/l	EDTA Titrimetric Method (APHA-2012)	0.01 N EDTA using Eriochrome black T
**Magnesium Hardness**	mg/l	EDTA Titrimetric Method	Magnesium hardness (mg/l) = (Total hardness–Calcium hardness) × 0.243
**Nitrate nitrogen**	μg/l	Acid per sulphate digestion	Spectrophotometer (Human Corporation, Japan) at 410 nm
**Sulphate**	mg/l	Indirect complex metric EDTA Titration method	Spectrophotometer (Human Corporation, Japan) at 420 nm
**Total phosphorus**	μg/l	Acid per sulphate digestion	Spectrophotometer (Human Corporation, Japan) at 690 nm

**Table 3:** Diversity index formulae used for selected three sites.

Index	Symbol	Formula	Source
Shannon-Weiner diversity index	H	**-** Σ *p*_*i*_ *(lnp*_*i*_*)*	Shannon and Weiner,1963
Pielou’s Evenness index	J	H / ln S	Pielou,1966
Jaccard’s similarity index	Cj	j/(a+b-j)	Jaccard,1912
Relative abundance	RA	n×100/N	Nagi and Mamgain,2013

S = Total number of species in the community (richness), *P*_i_ = Proportion of S made up of the one species,ni = Total number of individuals of each species in each sample, N = Total number of individuals of all species in the sample, J = Pielou’s evenness index,H = The observed value of Shannon index, Cj = Similarity between any two zones a and b, j = No. of species common to both zones a and b, a = No. of species at zone a, b = No. of species at zone b.

## 3. Result and discussion

### 3.1 Environmental factors

Environmental factors in the current investigation varied greatly on a spatiotemporal scale. Analysis of variance (ANOVA) revealed significant variation among the parameters between the sites and seasons. The resulting *F* values show the significance of observed variation of each parameter at 5% level of significance (Table 4). There is a statistically significant differences between sites as well as between the seasons (p <0.05) along the longitudinal gradient from site- I to site-III. Dissolved oxygen displays decreasing trend while other parameters show increasing trend from site-I to site-III. The mean values for the physicochemical characteristics at three sample sites (SI to S3) in the Vaishav stream over the period of one year (October 2020 to November 2021) were recorded. The highest water temperature was recorded in the summer at site III (10.33 ± 2.05), while lowest value was recorded in the winter at site-I (1.33 ± 0.47). The summer months generally attributes clear sky, long photoperiods, and higher solar radiation, the winter months have short photoperiods, lower solar radiations, and frigid air temperatures [28].

**Table 4:** Spatial and temporal variability of the environmental characteristics of sampling sites of Vaishav stream (*p* < 0.05).

Physico-chemical Variables	F	p-values	Ranking (Post Hoc Test)
**Air Temperature (** ^**o**^ **C)**			
**Between-subjects (Sites)**	**4.097**	**0.0362**	**Site-I, Site-II, Site-III**
Within-subjects (Seasons)	72.5	**0.000**	4 Seasons vs each other
**Water Temperature (** ^**o**^ **C)**			
Between-subjects (Sites)	3.074	**0.0389**	Site-I, Site-II, Site-III
Within-subjects (Seasons)	60.67	**0.000**	4 Seasons vs each other
**Turbidity (N.T.U)**			
Between-subjects (Sites)	49.938	**0.000**	Site-I, Site-II, Site-III
Within-subjects (Seasons)	3.916	0.0415	4 Seasons vs each other
**Electrical Conductivity (μS/cm)**			
Between-subjects (Sites)	5.686	**0.0253**	Site-I, Site-II, Site-III
Within-subjects (Seasons)	4.889	0.0261	4 Seasons vs each other
**TDS (mg/L)**			
Between-subjects (Sites)	15.972	**0.0015**	Site-I, Site-II, Site-III
Within-subjects (Seasons)	5.5444	0.0201	4 Seasons vs each other
**Ph**			
Between-subjects (Sites)	4.35	**0.0289**	Site-I, Site-II, Site-III
Within-subjects (Seasons)	3.022	**0.0472**	4 Seasons vs each other
**Dissolved Oxygen (mg/L)**			
Between-subjects (Sites)	77.538	**0.000**	Site-I, Site-II, Site-III
Within-subjects (Seasons)	4.077	**0.0401**	4 Seasons vs each other
**Free CO**_**2**_ **(mg/L)**			
Between-subjects (Sites)	7.65	**0.011**	Site-I, Site-II, Site-III
Within-subjects (Seasons)	3.7801	**0.0307**	4 Seasons vs each other
**Total Alkalinity (mg/L)**			
Between-subjects (Sites)	45.150	**0.0002**	Site-I, Site-II, Site-III
Within-subjects (Seasons)	4.1842	**0.0389**	4 Seasons vs each other
**Total Hardness (mg/L)**			
Between-subjects (Sites)	10.109	**0.000**	Site-I, Site-II, Site-III
Within-subjects (Seasons)	5.13	**0.0201**	4 Seasons vs each other
**Calcium Hardness (mg/L)**			
Between-subjects (Sites)	10.644	**0.0042**	Site-I, Site-II, Site-III
Within-subjects (Seasons)	4.189	**0.0422**	4 Seasons vs each other
**Magnesium Hardness (mg/L)**			
Between-subjects (Sites)	4.152	**0.0446**	Site-I, Site-II, Site-III
Within-subjects (Seasons)	4.897	**0.0399**	4 Seasons vs each other
**Ca**^**2+**^ **(mg/L)**			
Between-subjects (Sites)	10.64	**0.0042**	Site-I, Site-II, Site-III
Within-subjects (Seasons)	4.049	**0.0412**	4 Seasons vs each other
**Mg**^**2+**^ **(mg/L)**			
Between-subjects (Sites)	4.067	**0.0429**	Site-I, Site-II, Site-III
Within-subjects (Seasons)	8.876	**0.0059**	4 Seasons vs each other
**NO**_**3**_ **-N (μg/L)**			
Between-subjects (Sites)	3.56	**0.0470**	Site-I, Site-II, Site-III
Within-subjects (Seasons)	4.147	**0.0422**	4 Seasons vs each other
**SO**_**4**_^**2-**^ **(mg/L)**			
Between-subjects (Sites)	4.419	**0.0397**	Site-I, Site-II, Site-III
Within-subjects (Seasons)	9.57	**0.0054**	4 Seasons vs each other
**Total Phosphorus (μg/L)**			
Between-subjects (Sites)	35.411	**0.000**	Site-I, Site-II, Site-III
Within-subjects (Seasons)	4.228	**0.0400**	4 Seasons vs each other

At Site-III greatest turbidity value of (13.53 ± 0.41) was observed followed by least value of (1.54 ± 0.20). Earlier work Patra et al. (2011) also found fluctuating values of turbidity and a variety of human activities such mining, sewage disposal, and residential area effluent are possible factors associated with it. Higher electrical conductivity was observed at site-III (178.33 ± 4.18) in the month of April, whereas at site-I electrical conductivity found was (74 ± 4.32) in the month of January. The rise in electrical conductivity may be brought on by drainage into the stream, overuse of agricultural fertilizers, high nutrient enrichment, urban and agricultural land use, and stream bank erosion. Heavy rainfall in March and April causes the input flow to have a higher ionic content, which in turn raises the water [29]. The mean value of total dissolved solids ranges between 48.1 ± 2.80 mg/l to 115.91 ± 2.72mg/l, with site III having the highest value and site I having the lowest. The greatest value was recorded during the rainy season in April, while the minimum value of 62mg/l was recorded during the winter in the month of January. This is a result of snowmelt, strong rainfall, and rising runoff in the stream’s catchment region. Alkalinity is also increased by anthropogenic activities like as mining, sewage, agricultural runoff, and siltation brought on by surface run-off [30].

The pH varied from (7.06 ± 0.09) to (8.9 ± 0.43) at site III having the greatest value in the summer and at site I having the lowest value in the winter. Agricultural runoff, residential sewage, urban runoff, and commercial runoff from the catchment region may all be contributing factors to the summertime pH decrease [31]. The greatest mean DO value (13.16 ± 1.38) was measured in the upper stream at Site I throughout the winter, while the lowest value was 8.2 ± 0.71) downstream at Site III. Due to low temperature, strong photosynthetic activity, altitude, high turbulence, and little anthropogenic pressure, the Site-I maintained high DO throughout the experimental period. Water with little biological activity and low oxygen content can rapidly become saturated at low temperatures [32]. Low DO level at site-III is caused by an increase in temperature, organic matter, agricultural runoff, residential and municipal sewage, quicker breakdown of organic matter, and reduced stream flow [33]. Site-III recorded the greater free CO_2_ value (8.63 ± 0.28), while site-I recorded the lower value (1.5 ± 0.40). The rise in biological activity, waste water and sewage effluents, fertilizers, and other point and non-point sources from the agricultural fields are a result of the increasing value of free CO_2_ [34]. Low biological activity at site -I is the cause of the low carbon dioxide levels [35].

Site-III reported the greatest total alkalinity value (99.17 ± 11.496 mg/l), whereas site-I recorded the lowest value (52.00 ± 4.561). Water alkalinity is primarily caused by the formation of carbonates, bicarbonates, and hydroxides from the dissolving of carbon dioxide [36]. The high flow discharge might be because of the decreased alkalinity readings. Site-III had the highest total hardness, calcium hardness, and magnesium hardness, whereas site-I had the lowest total hardness. The data fall into three categories from (68.66±4.98 to 127 ± 2.44, 24±1.63 to 85.66 ± 2.05 and 6.88 ± 1.53 to 10.04±0.11mg/) respectively. Earlier Sharma et al., 2016 reported that Ca and Mg from beneath ground fluids contribute higher hardness [37].

The lowest concentration of nitrate (97 ± 5.71) was discovered at site I, and the highest value, 211 ±5.71 was discovered at site III. Due to loading from non-point sources, such as agricultural regions that are supplemented with nitrate-containing fertilizers, there is a greater concentration of nitrate [38]. Site-III had the greatest mean value of sulphate concentration 10.56 ± 0.46, whereas site-I had the lowest mean value 3.7 ± 0.66. Many factors influence the sulphate content in stream water, including the presence of bed rock in the catchment, the contribution of pedogenic sulphate, and desorbing sulphate, primarily of human origin [39]. The growing trend of sulphate downstream might be attributed to the discharge of sulphate ions from domestic and commercial sewage, as well as the increased usage of fertilizers in the downstream [40,41]. Similarly, the mean lowest value of total phosphrous 76 ± 1.63 was identified at site-I and the highest value of 270 ± 10.70 was reported at site-III. The rising downstream trend might be attributed to runoff from agricultural areas laden with dissolved fertilizers, urban runoff, and organic waste from domestic and municipal sewage [31].

### 3.2 Fish composition

**Fig 2:**
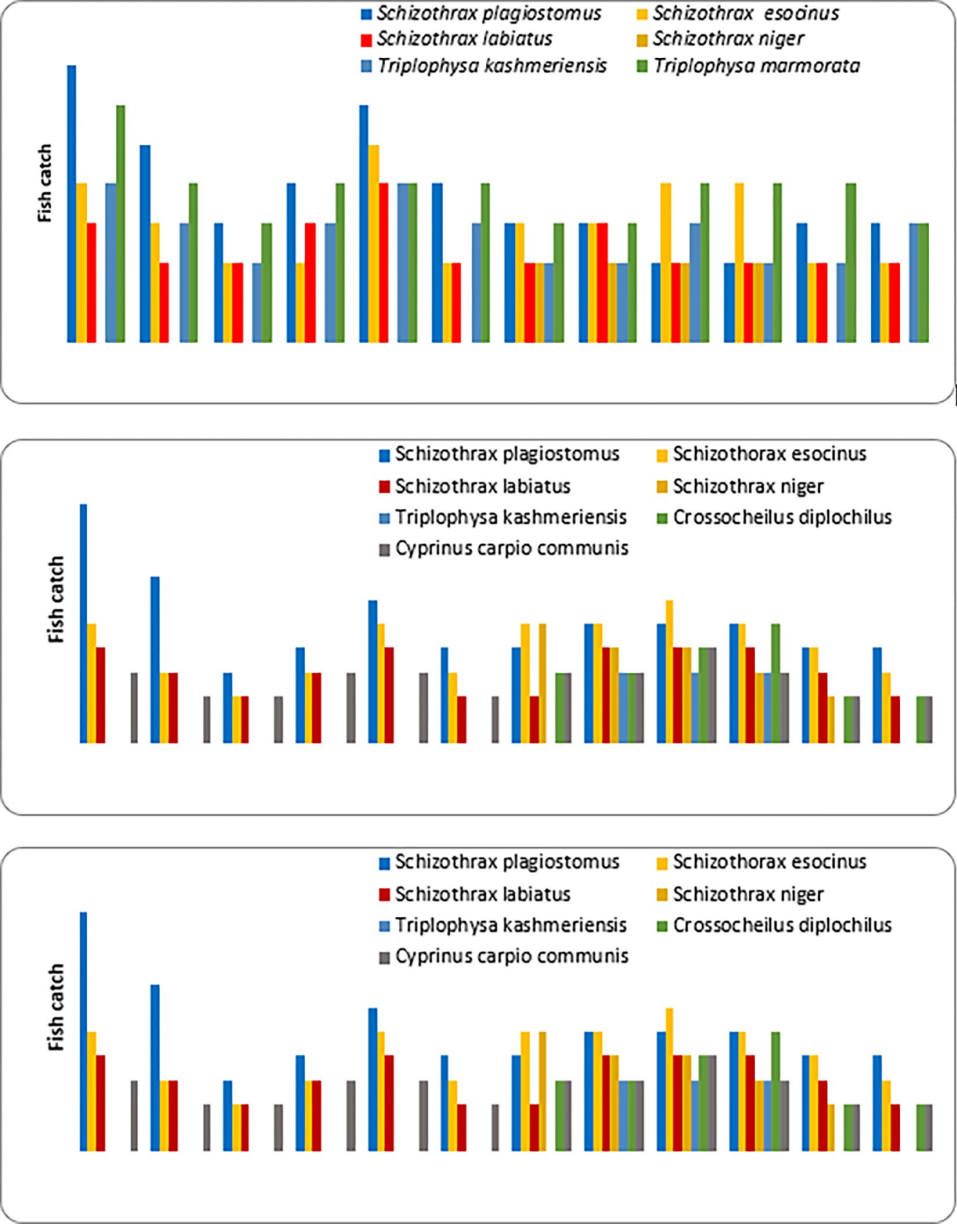
Monthly fish catch from Vaishav stream November 2019 –Oct. 2020 at Site-I, Site-II, and Site-III.

The biodiversity of Himalayan rivers encompasses high degree of endangerment and endemism [42]. Despite the fact that it has not received as much attention as in other parts of the world, particularly from the temperate rivers of European and North American nations, [43]. Habitat degradation is typically a result of anthropogenic activity and growing economic growth, which also degrades the water quality of riverine ecosystems [44]. In the present study we observed the diversity (richness) and fish community composition along the longitudinal gradient of Vaishav stream form the three sampling study sites. A total of 630 specimens belonging to 11 fish species, three orders *Cypriniformes*, *Siluriforms* and *Salmoniformes* and four families including *Cyprinidae*, *Nemachelidae*, *Siluridae* and *Salmonidae* were reported from the study sites. Among collected specimens, *Cypriniformes* were dominant with nine species followed by order *Siluriformes* and *Salmoniformes* with one species each. Out of eleven fish species, six fish species belongs to family *Cyprinidae*, three to *Nemachelidae*, one to *Siluridae* and *Salmonidae* each. In addition to this the monthly variation in fish catch from three sampling sites is depicted in (Fig 2). Which depicted the monthly abundance of collected fish species at three different sites along the stream gradient and *Schizothrax*.*plagiostomes* was the most dominated fish species recorded and was collected in every month throughout the year due to due to its adaptability to fluctuating environmental conditions and diverse diet of benthic algae and detritus. This flexibility allows it to thrive despite seasonal changes and anthropogenic pressures [45–47].

At Site-I (Watoo Reshinagar) *Schizothorax curviforns* was found to be most dominating fish followed by *Triplophysa marmorata*, *Schizothrax plagiostomus*, *Glyptosternon reticulatum*, *Oncorhynchus mykiss*. At Site-II (Kulgam) *Triplophysa marmorata* was found to be most dominating fishfollowed by *Schizothrax plagiostomus*, *Schizothrax esocinus*, *Triplophysa kashmirensis*, *Schizothrax labiatus*, *Schizothrax niger*. At Site-III (Arwani) *Schizothrax plagiostomus* was found to be most dominating fish followed by *Schizothrax esocinus*, *Schizothrax labiatus*, *Cyprinus carpio communis*, *Crossocheilus diplochilus*, *Schizothrax niger*, *Triplophysa kashmirensis* (Table 4). The dominance and the distributional pattern of fish community in the Vaishav stream showed spatial-temporal variation at three selected sites and the dominance pattern of the species by number followed the trend as *Schizothrax plagiostomus> Triplophysa marmorata> Schizothrax esocinus> Schizothrax labiatus> Schizothrax curviforns> Triplophysa kashmirensis>Cyprinus carpio communis>Glyptosternon reticulatum> Schizothrax niger>Oncorhynchus mykiss> Crossocheilus diplochilus* (Table 5). The percentage of fish population showed that order Cypriniformes was most dominant constituting 92.17% followed by order Siluriformes constituting 4.44% and order Salmoniformes constituting 3.33%.The minimum richness of (5) and abundance (208) was recorded at site-I and 7 and 228 at site-III respectively which increased from site–I to site-III, while seasonally minimum in winter 49 and maximum in summer 62 at site I. At site-II minimum in autumn 42 and maximum in summer 54likewise at site -III minimum in spring 42 and maximum in summer 79 (Table 5). Similar results were obtained by [14,48], while exploring the fish diversity on the Vaishav stream [14]. Similary, [49] reported seven fish species from Lidder stream viz, *Schizothorax plagiostomus*, *S*. *labiatus*, *S*. *esocinus*, *Salmo trutta fario*, *Crosscheilus diplochilus*, *Glyptostern reticulatum, Triplophysa kashmirensis [49]*. The fish species occurrence and relative abundance are influenced by a variety of variables, which includes water flow, water depth, water temperature, stream length, substrate type, food availability, and water quality characteristics [50]. *Glyptosteron reticulatum* is a hill stream catfish that is mostly confined in the upper stream (site-I) having some structural adaptive modifications, which helps the fish to adhere to the large stones in the torrential streams. The lips of the fish have adaptive modifications in scraping food entangled with the stones, which helps the fish to survive and flourish well in the hill stream so it is restricted to upper streams only. Furthermore, the fish prefers to live in rivers, tiny streams, and rivulets that are clean in the mountain and foothill areas [49,51]. This fish prefers to live in fast-moving areas of the river with stony terrain. They are, however, unable to fight the current of muddy torrents and are thus drifted downstream. Moreover, the fish during the daytime hide under stones and boulders. It is nocturnal and search food within a restricted station during the night. Similarly, *Oncorhynchus myksis* an exotic rainbow trout was introduced by the Department of Fisheries J&K (UT) in the Vaishav stream with the aim to propagate the trout in natural habitat and as contributed to the catch in most of the summer months of the year in the upstream at Station-I. This is related to the cleanness of the upstream water, where the high dissolved oxygen, moderate temperature, low nutrients load and high density of benthic insects. The trout being carnivorous in habit feeds on the benthic fauna, which flourishes well in the upper reaches (Site-I) which is the reason for the trout being restricted to upper zones only. Likewise, *Schizothrax curviforns* also prefers to live in swift, clean and oxygenated water that may be the reason for its presence only at site-I, in contrary to other *Schizothrax* species. Moreover, this trout fish unlike *Glyptosteron reticulatum*,is inherently inclined to move against the water current and therefore found in upper sites only. The trend is contrary to station (site-III) for small sized fishes like *Triplophysa kashmirensis*, *Crossocheilus diplochilus* and *Cyprinus carpio communis*. The availability of these smaller fishes in the upstream is scarce because of the inability to withstand the high current velocity and therefore these fishes are flushed downstream. The finding is also supported by the works of [52,53] who have reported that small fishes remain restricted to shallow stream margins as compared to upstream reaches, where the current is fast or too deep or both, [52,53]. Non-cyprinids such as Balitorids, according to [54], are mainly found around pool margins and in shallow waters [54]. *Cyprinus carpio communis*, an exotic carp was also introduced by the Department of Fisheries J&K (UT) in the rivers and streams to meet the demand of fish supply because of the characteristic feature of surviving in downstream only, where the water current is very low leading to the stagnant and suspended food being a coloum feeder. The diversity increases downstream by increase in channel stability, heterogenous substrate, food source, nutrients and stream water temperature [55]. In our study, we reported *S*. *plagiostomus* as the dominant species, followed by *Triplophysa marmorata* and *S*. *esocinus* respectively. Earlier research by [41], Vaishav stream has a comparatively low pollution load, and as a result, the 11 fish species appear to be very little environmental stress [41]. This has led to the almost ideal growth pattern of fishes in the river. [14,49] have reported *S*. *plagiostomus* the most dominant fish species occurring consistently from upward to downstream reaches, followed by *S*. *esocinus* and *Triplophysa kashmirensis [14,49].* [56] have reported higher abundance of *S*. *plagiostomus* to be significantly explained by the low conductivity and lowest value of water turbidity [56]. Physical habitat and water quality attributes are known to be vital factor for fish abundance [57]. Other environmental factors including dissolved oxygen and water temperature with increasing altitude across small geographical distances, impacts fish species distribution and richness. As per the work of [58], most fish communities decline as altitude increases, but not all fish spp. respond to altitude in the same pattern with trends peaking at mid and high elevations [59]. The decrease in niche space and the increasing severity of climatic conditions are typically known for the limited variety in high altitude areas. On the other hand, at low-altitude areas with moderate environmental conditions and complex habitat seems to have great impact on diversity and richness. Other environmental variables, such as the regional climate and anthropogenic activity might disturb the altitudinal gradients [60]. The sites characterized by slow-flowing waters become favorable for the sustenance and growth of eurytopic or stagnophilic fishes [61]. The seasonal fluctuations in flow and discharge during the monsoon in rivers disturb the aquatic ecosystem by displacing the aquatic life [62].

**Table 5:** Seasonal Occurrence of fish species sampled in three stations of Vaishav Stream (J&K), 2019–2020. Probable seasonal Occurrence (S.O. and site occurance, column) indicated by symbols: Y- Yearlong occurrence, W- present only warmer months, e- entire stream. Sampling with the help of fishermen.

Fish species	Nov.	Dec.	Jan.	Feb.	Mar.	Apr	May	June	July	Aug.	Sept.	Oct.	Total	S.O.	Site
*Schizothrax plagiostomus*	22	16	09	11	17	11	10	12	12	11	10	09	**150**	Y	E
*Schizothrax esocinus*	09	06	04	05	10	05	08	08	10	09	06	05	**85**	Y	II & III
*Schizothrax labiatus*	07	05	04	06	08	04	04	07	06	08	05	04	**66**	Y	II & III
*Schizothrax curviforns*	07	06	04	04	07	04	05	05	06	05	03	03	**59**	Y	I
*Schizothrax niger*	-	-	-	-	-	06	05	06	05	04	-	-	**26**	W	II & III
*Triplophysa kashmeriensis*	04	03	02	03	04	03	02	02	03	02	02	03	**42**	Y	II & III
*Triplophysa marmorata*	12	08	07	08	08	08	07	07	09	09	10	09	**102**	Y	I & II
*Glyptosteron reticulatum*	02	02	02	02	03	02	03	03	03	02	02	02	**28**	Y	I
*Oncorhynchus mykisis*	-	-	-	-	-	-	03	04	05	05	04	-	**21**	W	I
*Crossocheilus diplochils*	-	-	-	-	-	-	03	03	04	05	02	02	**19**	W	III
*Cyprinus carpio communis*	03	02	02	03	03	02	03	03	04	03	02	02	**32**	Y	III

**Fig 3:**
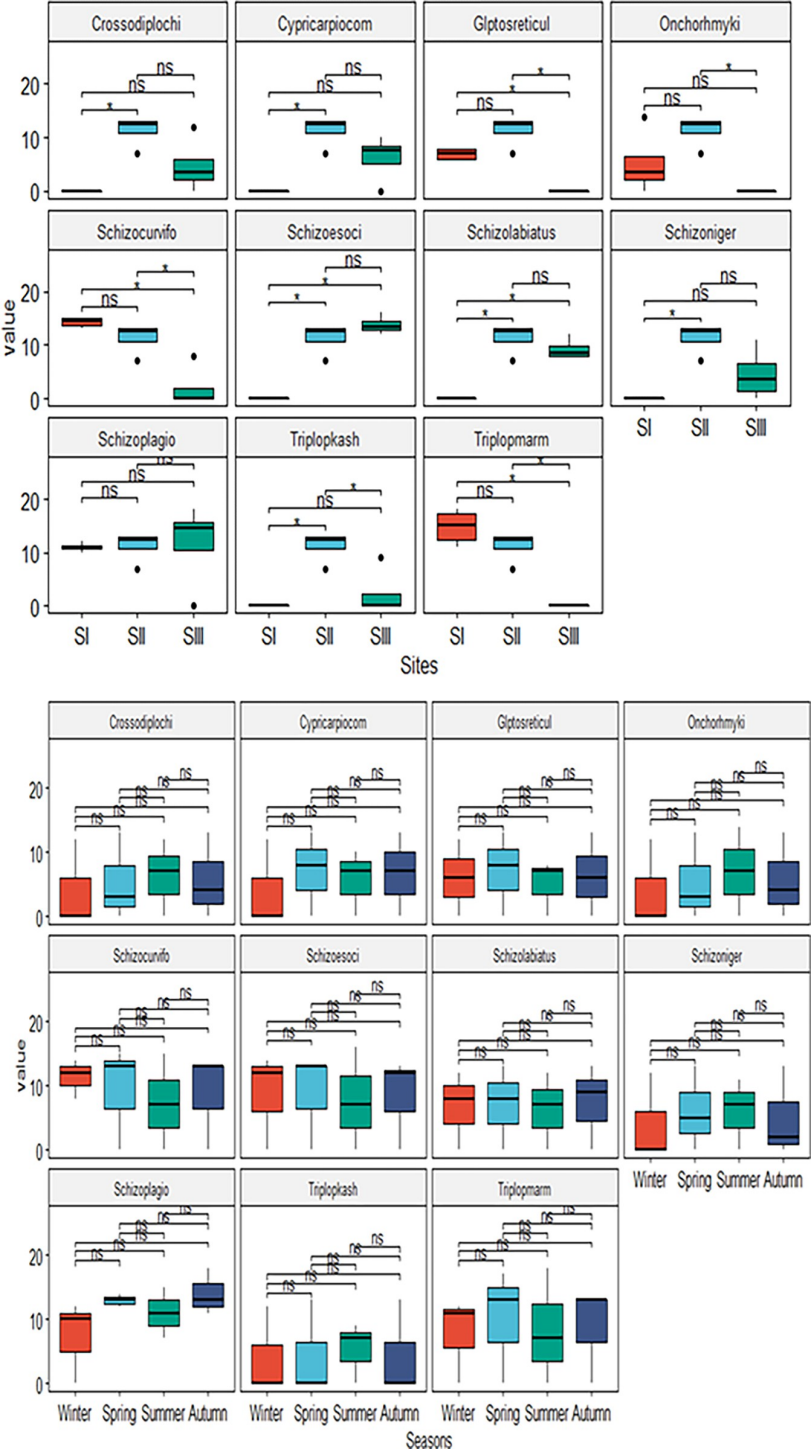
Illustration of t-test between the sites and between the seasons, ("***" = 0.001,"**" = 0.01,"*" = 0.05, ns = not significant).

**Fig 4:**
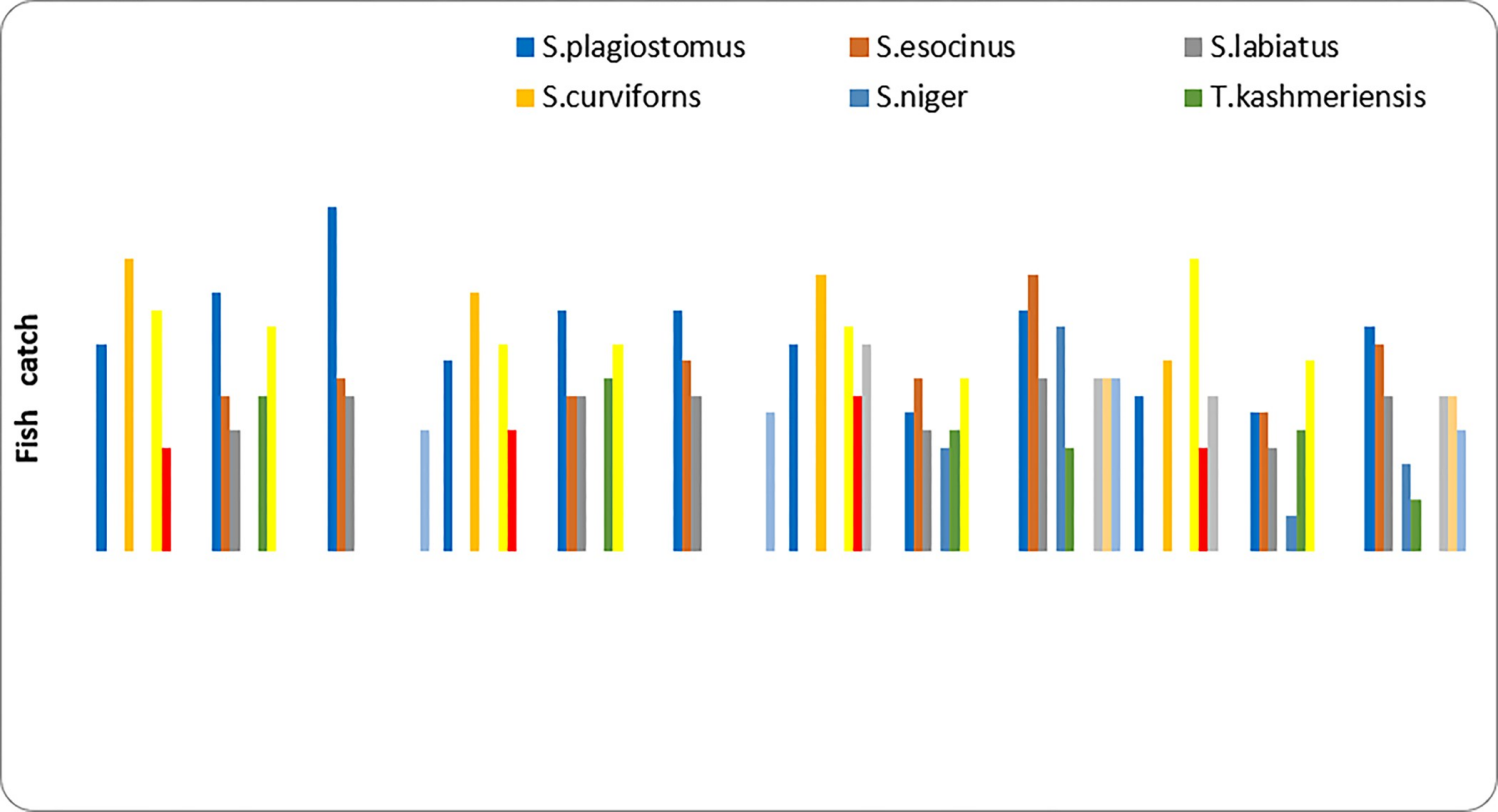
Seasonal fish catch in Vaishav stream at three selected site from Nov. 2019—Oct. 2020.

On a seasonal basis, all species were collected in four seasons including winter, spring, summer and autumn. About 8 eurythermal species indicated by Y column in Table 4 were collected in all seasons at two sampling sites. Additional 3 stenothermal species were collected in warm months only. Our results indicate that fish abundance across the site s (I, II & III) were significant but not among the seasons (winter, spring, summer & autumn) (Figs 3 and 4). Most findings have observed significant spatial and temporal variations in fish abundance in natural streams [63]. Spatial variations along the longitudinal gradient in fish abundance were usually caused by changes in habitat features and seasonal variability in fish abundance was often attributed to floods which cause seasonal migration of fish species [64].

In our study, we found that fish abundance of the Vaishav stream varied significantly along the longitudinal gradient (spatially) with increasing stream order from site-I to site-III, However, no substantial variations in fish diversity was seen between the seasons, which was consistent with the studies of [65,66]. Moreover, these findings together imply that temporal changes. Likely, fish abundance in these streams were decided more by spatial heterogeneity in environmental conditions than seasonal variation [67].

### 3.3 Diversity indices

Diversity indices were determined to understand the concept ecology of a community and their interaction with one another [68]. Site-III has highest alpha diversity in contrast with site-I. From alpha diversity metrics, it becomes clear that site–III is differently structured. Shannon diversity is a widely used metric for assessing diversity in different ecosystems [69]. The Pielou Evenness Index measures how evenly individuals are distributed among the sites and low evenness means only few species dominate the site [70]. The diversity index values in the three stations and all indices are ranked as the most and least diverse respectively. Jaccard’s index showed more similarity in fish fauna between site-II and site-III than site-I and site-III (Table 6). Site-III (Arwani) has the highest fish species richness followed by Site-II (Kulgam) and Site-I (Watoo Reshinagar). The Site-II (Kulgam) and Site-III (Arwani) had the highest Shannon H’, and Pielou Evenness index value, while the Station Site-I had low Shannon value H’ and Evenness index value (Watoo Reshinagar). Shannon-Weiner index (H) value of 1.48 and Pielou Evenness (E) value of 0.82 was obtained by [14] indicating that diversity of fish is very less in Vaishav stream [14] and is dominated by few species i.e. evenness is poor among species. The low species richness (Shannon H) at site I may be due to slightly harsh in stream environmental conditions such as low temperature high flow and current/ velocity and less suitable habitat area for proper growth and development, moreover, environmental filtering have allowed few species to dominate the site there thereby limiting their distribution and consequently influencing the population composition [59]. The low fish diversity at Station-I may be attributable to the higher altitude (2266 masl) compared to Station-II (1882 masl) and Station-III (1534 masl) [71], as well as the fact that species diversity rises with stream order (Bhat, 2013). The higher fish diversity Shannon H’, in lower reaches (site-III) may be due to low altitude, moderate water quality conditions, diverse substrate, ambient temperature, riparian vegetation, flow and velocity providing refugee to the organisms increases [58,72].

**Table 6:** Percentage (%), total abundance and relative abundance of fishes in Vaishav stream (J&K), at three selected stations from Nov.2019 –Oct. 2020. Indicated by symbols: TA- Total dominant, RA- Relative abundance, D- dominant, M- moderate, R- rare.

Fish species	Site-I			Site-II			Site-III			Total	%	Status
	%	TA	RA	%	TA	RA	%	TA	RA			
*Schizothorax plagiostomus*	21.15	44	26.82	22.16	45	28.48	27.11	61	37.19	**150**	23.80	D
*Schizothorax esocinus*	_	_	_	17.73	36	21.55	21.77	49	33.56	**85**	13.49	M
*Schizothorax labiatus*	_	_	_	14.28	29	16.66	16.44	37	19.68	**66**	10.47	M
*Schizothorax curviforns*	28.36	59	39.59	_	_	_	_	_	_	**59**	9.36	M
*Schizothorax niger*	_	_	_	3.94	08	4.10	8	18	8.69	**26**	4.12	M
*Triplophysakashmirensis*	_	_	_	16.25	33	19.41	4	9	4.16	**42**	6.66	M
*Triplophysa marmorata*	26.92	56	36.84	22.66	46	29.29	_	_	_	**102**	16.19	M
*Crossocheilus diplochilus*	_	_	_	_	_	_	8.44	19	9.22	**19**	3.01	R
*Glyptosteronreticulatum*	13.46	28	5.55	_	_	_	_	_	_	**28**	4.44	M
*Oncorhynchus mykisis*	10.09	21	11.22	_	_	_	_	_	_	**21**	3.33	R
*Cyprinus carpio communis*		_	_		_	_	14.22	32	16.58	**32**	5.07	M
**Total**										**630**	100	

In the present study, we observed that fish species richness increased from upstream to downstream due to altitude difference which is believed to be the main cause that influences the fish community composition in rivers and streams. Further some other factors which increase the fish diversity include habitat and food, stream order [73]. Hence longitudinal pattern of fish distribution are controlled by a number of factors like site elevation, water temperature and stream size & width [74]. This trend seems to be associated with species diversity as indicated in streams, where channels are wide, unshaded [75]. Among the sampling sites we found the marked difference in diversity, abundance, and distribution of fish species. These differences are primarily due to the habitat conditions and secondarily due to the influence of environmental factors such as anthropogenic pollutants, and relative tolerance of the fish species at the sampling sites. Furthermore, fish diversity from downstream was high, having pool habitats [30]. Freshwater fish diversity is reported to be quite high in low and intermediate land locations as deep water bodies allow niche segregation allowing fish to exist without being subjected to increased intra and interspecific competition [76]. However, when the water temperature and volume dropped in the winter, there were fewer and fewer diverse habitat and food supplies available [77]. Due to the somewhat warmer water temperatures and abundant food supplies, many species have a tendency to colonies wider and deeper habitats under this environment [78]. According to research, the riffle environment supports fewer species than pool habitats due to changes in water temperature, strong flow/velocity, and a lack of available food resources. However, during the dry season, when there is little water flow or volume, several fish species become stranded in tiny, shallow ponds. According to the research done by [79], the sample sites (S-I and S-II) were predominated by rocks, fine sediments, and gravels, but the downstream portions of the river (S-III) were prevailed by suspended silt, sand, clay, and macrophytes [79]. Hubbel (2001) also observed that the main geographical barriers viz. forests, mining, stones and bridges could be the possible reason for limiting the movement of fish in the water [80]. During the dry season (autumn) when the water level decreased the fishes move from upward to downwards and migrate back to upwards during the rainy season (spring). Lim et al. (1999) reported that decrease in water level causes the migratory fish species to move from the lake to the Mekong River, and they migrate back to the lake at the beginning of the rainy season [81]. The migration of fish appeared to be the main factor that resulted in the high amount of fish collection in the confluence area of the stream with Main River may increase the richness [82]. Therefore, these findings justify our collection at site-III with high species diversity and collection.

### 3.4 Community similarity (Jaccard’s index)

In ecology, Jaccard’S index is used to measure the similarity or disimilarity by comparing species composition between different sampling sites of ecological communities. During the present it was observed that Site-I and Site-II have 22% of similarity whereas Site-II & Site-III have 62% of similarity and 9% similarity have been shown by Site-I & Site-II (Table 7). The 22% similarity between Site I and Site II suggests limited species overlap, likely due to differences in habitat structure and water quality. Site I, a cooler, oxygen-rich headwater, predominantly supports rheophilic species, while Site II has altered habitat and physico-chemical conditions. Headwaters typically host unique assemblages due to distinct environments compared to midstream areas [83]. he 62% similarity between Site II and Site III reflects a significant overlap in species composition, likely due to their proximity and similar environmental conditions. Shared flow regimes, substrate types, or human impacts may contribute to a more uniform fish community, especially in mid- to downstream areas where conditions are typically more consistent [71]. The 9% similarity between Site I and Site III highlights their distinct ecological conditions, with Site I influenced by headwater characteristics and Site III affected by downstream nutrient input, sediment load, and human activities. Similar studies [21] show significant fish composition differences between upstream and downstream sites due to varied environmental stresses and habitat fragmentation. Moreover, streams have experienced significant changes from various human activities, which also impact species migration between river habitats [84,85].

**Table 7:** Jaccard’s similarity index of fish species at three selected sites.

Site	I			II			III		
	JS	PS	JD	JS	PS	JD	JS	PS	JD
**I**	-	-	-	0.22	22%	78	0.09	9%	91
**II**	0.22	22%	78	-	-	-	0.62	62%	38
**III**	0.09	9%	91	0.62	62%	38	-	-	-

### 3.5 Cluster analysis

**Fig 5:**
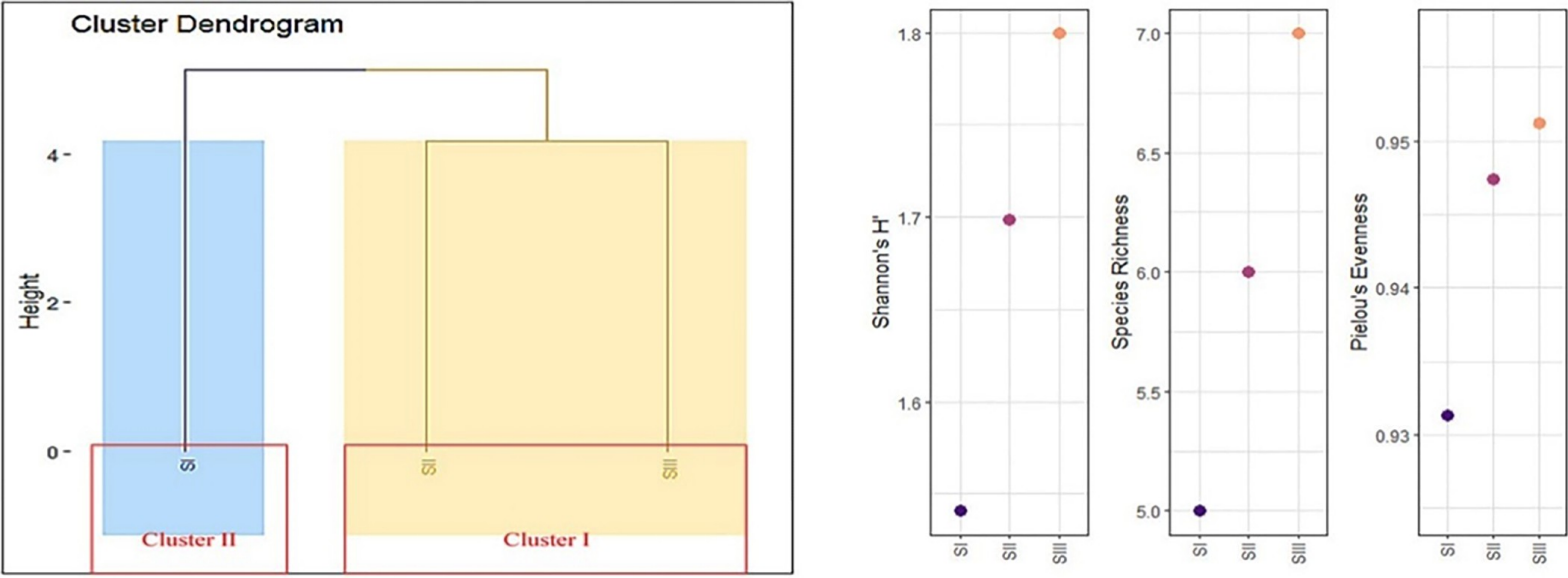
Cluster dendrogram and alpha diversity between the sampling sites on the basis of fish community.

Clustering was applied to determine the similarity between the sampling sites on the basis of fish community. Fish community datasets obtained from three sites of Vaishav stream was subjected to cluster analysis, a powerful data tool for extracting information from multivariate data sets. The goal is to identify groups (clusters) of similar objects in a given dataset of interest. Cluster analysis indicated two major groups in fish assemblage showing a similarity with each other. In general, samples from site 1 maintained a similarity with site II, as these sites showed some similarity as they were situated close to each other and had roughly similar environmental parameters whereas the site III showed quite a dissimilarity with respect to site I and II as it was located farther with varied environmental conditions. Although their amount of contribution varied, the major contributing species in the sites were more or less comparable. The main driver behind similarity and dissimilarity was seasonality, as it changed hydrological conditions and thus impacted the fish fauna [86] (Fig 5).

### 3.6 Fish community pattern

**Fig 6:**
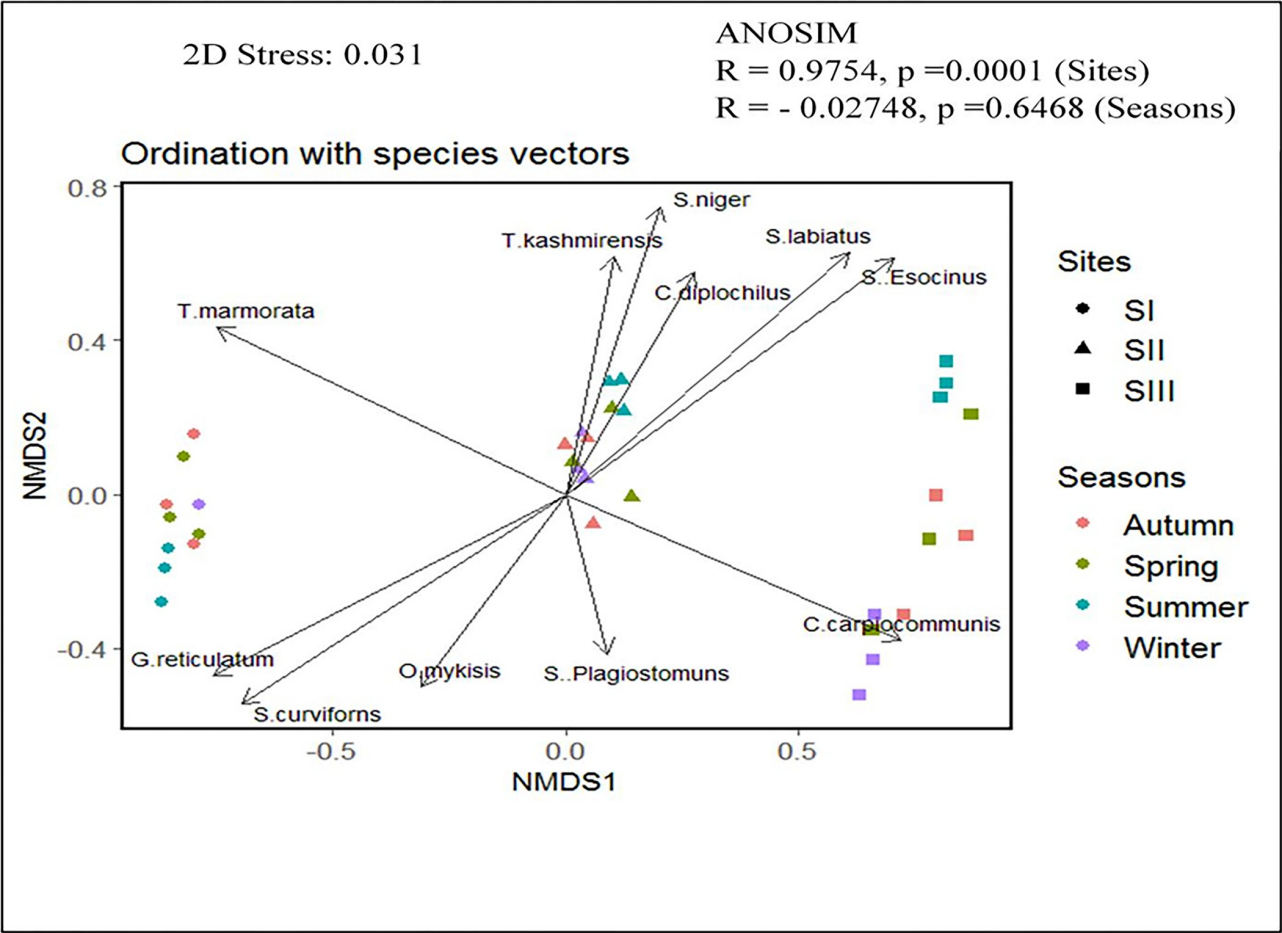
NMDS showing spatiotemporal patterns and relationships between various environmental factors and fish community.

NMDS based on fish abundance (count) data (stress = 0.031) followed by ANOSIM (*p*< 0.05) revealed a clear distinction based on sites (R = 0.9754, p = 0.0001), while the difference based on seasons was not statistically significant (R = -0.02748, P = 0.6468) with statistical significance carried out at 9999 permutations (Fig 6). It was obvious from the research that there was no distinct separation of sites depending on seasonality, indicating that taxa’s preferences for certain locations did not change with the seasons. Permutational analysis of variance (PERMANOVA) test indicated strong evidence in favour of the null hypothesis of significant difference among sites based on count data. Similar findings on fish richness in mountain streams of the Ren River, southwest China, were made by [85], who also concluded that there was no discernible seasonal variation in the communities of fishes studied. Significant overlap between fish assemblages in wet and dry seasons was evident in NMDS ordination plots, and ANOSIM further indicated that there was no evidence of seasonal variations in the fish assemblages (R = -0.022, p = .745 >.05). Based on abundance data, the fascinating interactions between sites and seasons show that regional landscape features have a stronger impact on the quantity and composition of fish. However, in comparison to site-wise differences, seasonal differences were not as strong as those based on abundance data. Aquatic vertebrates vary less seasonally despite substantial spatial variation in fish composition and abundance, which may be related to reduce inter annual replacement of fish with seasonal habitat changes, difficult in-stream circumstances, behavioural, and life history changes [87,88]. Variations in flow, precipitation, isolation, and climatic conditions have a significant impact on aquatic fauna’s reproduction, growth, emergence timing, and development [89]. Middle sites (SII) are characterized by moderate anthropogenic sources in the near watershed, whereas upstream sites (SI) are well within the forest region and have little anthropogenic activity. High levels of horticultural and agricultural activity, human settlement, and stream bed excavation are features of the downstream reaches (SIII). Due to the existence of fish species that are essentially identical, SII and SIII may overlap, but SI is distinguished by the presence of fish species that are sensitive to pollution and those found in hill streams. The form and content of aquatic communities are significantly impacted by the worsening of water quality brought on by changes in land use and human activity [90]. Studies of [65] indicate spatial components of variation in fish assemblages in the upper Brazos River were greater than seasonal components [65]. [64] found that fish assemblages in Meridional Amazonian streams changed among watersheds (spatial variation), but not seasonally (temporal variation). Fish assemblages in Northwestern Great Plains streams varied more spatially than temporally [66]. Similarly, [91] observed that fish assemblages in the Puxi Stream were significantly different in spatial variation but not in temporal variation [92]. [93] found that fish assemblages in a mountain stream of the north Tiaoxi River differed along the stream continuum, but there was little apparent change associated with the seasons [93]. These studies together suggest that seasonal variations in habitat features may not always lead to temporal changes in the structure of fish assemblages. One of the possible reasons seems to be that fish assemblages in these streams were determined more by average or persistent spatial heterogeneity in environmental conditions and environmental variability than by seasonal variation in environmental conditions [65,66].

### 3.7 Principal Component Analysis (PCA)

**Fig 7:**
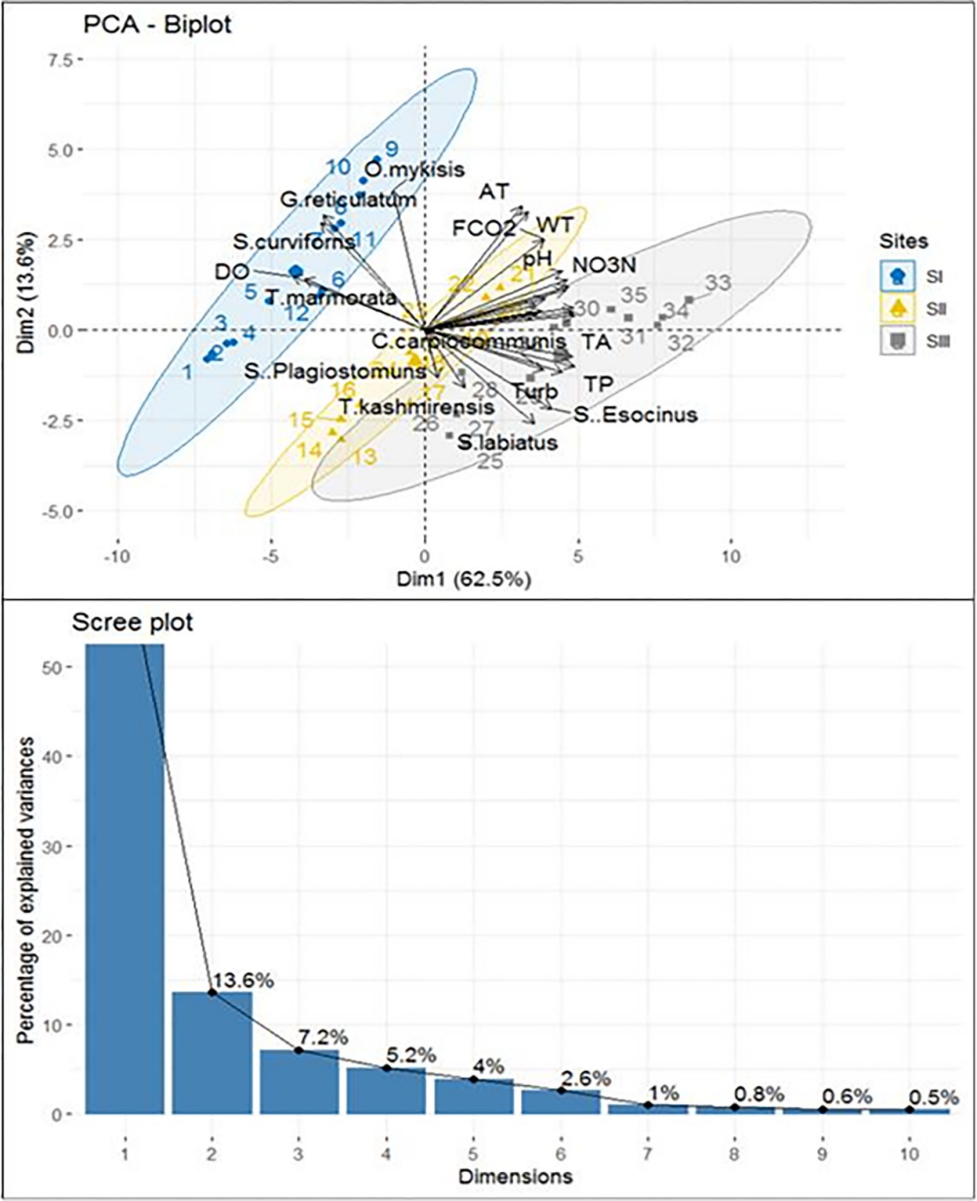
Principal component analysis and scree plot between fish components and environmental variables.

Kaiser-Meyer-Olkin (KMO) and Bartlett’s sphericity test were used to assess the suitability of the datasets for FA before PCA analysis. KMO (0.76) and Bartlett’s sphericity result (1345) both indicated that the dataset is suitable for PCA at p<0.05 (Lo et al. 2012). A dataset with a large number of linked variables is reduced in dimension using PCA. To do this, the dataset is divided into a new set of variables called principle components (PCs), which are orthogonal (non-correlated) and are organized in decreasing order of relevance [94]. Water quality data was normalized by Z-scale transformation to reduce classification errors caused by the high degree of dimensionality in the data [95]. Normalization was done to comply with the presumption of normality for statistical analysis (minimising the effects of various measurement units and parameter variances) [96]. The PCA analysis was executed using “prcomp” function and “ggbiplot” package. The statistical analyses were carried out using R Programming Language. PCA was executed on the whole water quality and fish datasets to recognize the basic environmental mechanisms driving the observed distribution of fish community. The factor loadings with values > 0.75, from0.75–0.50 and 0.50–0.30 are considered to be of high, moderate, and weak magnitude, respectively. PCA of the spatial clusters dataset yielded two principal components with eigenvalues 17.5 and 3.80 (PCs) explaining 76.11% of the total variance (Fig 7).

The PC1 explained 62.15%of the total variance, with strong and moderate positive component loading for, *S*. *Esocinus*, *C*. *diplochilus*, *S*. *niger*, *S*. *labiatus* and *C*. *carpio communis*, WT, Turb, Cond, TDS, pH, FCO_2_, TA, TH, CH, MH, NO_3_N, TP and SO_4_, and strong negative loading from dissolved oxygen and *T*.*marmorata*, *G*. *reticulatam* and *S*. *curviforns*. The PC2 explained 13.6% of the total variance, with strong component loading from, AT, WT, *O*. *mykisis*, *T*. *kashmirensis* and *S*. *curviforns* and strong and moderate negative loading from *S*. *esocinus*, *S*. *labiatus* and *T*. *kashmirensis*.

The PC3 explained 9.8%of the total variance, with strong positive component loading from, NH_3_N and NO_3_N and negative loading from DO. The PC4 explained 7.9%of the total variance, with strong and positive component loading from, WT and, NO_2_N.

### 3.8 Fish abundance and environmental factors

Earlier studies on riverine ecosystem have documented the prominent environmental factors responsible for the distribution and abundance of macro-vertebrates [97]. And the significant factors responsible for controlling the fish diversity (including habitat heterogeneity, substrate discharge /flow, velocity, temperature, elevation, stream size, riparian vegetation) and other chemical variables at varying saptio-temporal scales [98–100]. The higher anthropogenic activities result in higher concentration of turbidity, conductivity, total dissolved solids and substantial decrease in dissolved oxygen levels, resulting in lower species richness. Fish abundance and diversity are highly associated with dissolved oxygen, velocity, pH, conductivity in river water [57]. In recent years, studies at global level have shown that few freshwater fish species are competent to alter their regular distribution pattern in response to climate change. The quality of water directly or indirectly affects the fish diversity, distribution and production [101]. Change in river hydrology and connectivity had disrupted the longitudinal migration of fishes in Rivers across the globe [102]. Freshwater fishes are sensitive to environmental variation as they required optimum range of environmental variables and their alteration could be detrimental to them.

Due to seasonal fluctuations in environmental factors like temperature and dissolved oxygen, alters the composition and relative abundance of fish community [103]. The fish composition and distribution in fresh water ecosystem have been influenced by the concentration of physicochemical parameters such as pH and dissolved oxygen are essential for the growth and survival of fish fauna [104]. The influence of environmental variables on fish species distribution and community structure contributes to a more complete understanding of fish habitat relationships. [105] reported that the dissolved oxygen and turbidity significantly affect the fish distribution and composition along with some anthropogenic factors which seriously devastated fish habitat resulting in the decline of fish diversity in Ganjiang River China [105]. In the present study it was observed that water temperature increased longitudinally from upstream to downstream sites. pH was also influential in explaining the presence or absence of fish species. The optimal pH for freshwater fish species usually ranges from 5.5to 7.5 [15], which is consistent with the results of our study. Water, temperature, pH, conductivity, dissolved oxygen, and turbidity have been observed to influence the fish diversity composition and distribution in river Basin, Iran [106] and in Khandbari Municipality in Sankhuwasabha District, Eastern Nepal [107]. Likewise, EC, DO, pH, and alkalinity, were most strongly correlated with fish community composition of the Kali Gandaki River basin in Nepal [108], and the Ganga River basin in India [109,110]. EC and DO were influential factors in tropical streams in India Our results concur with the findings from these studies and further support the importance of these environmental variables in characterizing fish environment relationships. While studying the influence of environmental factors on fish abundance and distribution on River Singhiya Nephal it was observed that water temperature, pH, hardness have a significant relationship with fish diversity [111]. [105] reported that the dissolved oxygen, and turbidity significantly affect the fish diversity, distribution and composition in ‘Ganjiang’ river China [105]. While studying the relationship of water quality parameters with fish abundance and richness it was revealed that dissolved oxygen, total hardness, alkalinity, electrical conductivity, and TDS have significantly influenced the fish abundance and distribution.

Furthermore, while studying the fish diversity and its relationship with environmental variables in Kamala River, Nepal it was revealed that the species variation in relationship with environmental parameters like, temperature, electrical conductivity, TDS, and Nitrate were also found significantly correlated [112]. A study was carried out to investigate the variation of water quality parameters and their correlation with fish abundance and diversity in Bhini stream, A tributary of River Ravi in Jammu and Kashmir, India observed that TDS and nitrates were significantly correlated with the species diversity and abundance [104]. Similar studies of water quality parameters in relation with fish abundance was carried in Tropical Savanna Headwater streams it was observed that water temperature, conductivity, and dissolved oxygen helps in shaping the fish structure and assemblage [57] which complements with our results.

## 4. Conclusion

Diversity indices allow for the summation of information about a population’s numerical structure. In the current study, we found differences in species diversity at sampling stations which indicates that disturbed ecosystems support few species, whereas least disturbed niches are characterized by a large number species. Further, Himalayan ecosystem is fragile and thus it is very important to understand its biodiversity from conservation and management perspective which is still not well documented for various taxonomic groups including fishes. Therefore, each and every study which covers biodiversity of Himalayan region becomes very important. The present study shows various biodiversity indices and distribution pattern of fishes in Vaishav stream of Kashmir (India) Himalayas. In addition, the results of this study will aid in the planning and management of sustainable fisheries and natural resource protection at the national level. Much more research is required in order to build a strategy for the general enhancement of the region’s fishing resources. This study was formulated to determine the spatial-temporal variation in fish community structure in relation to the varying environmental attributes and also provides an inclusive understanding of water quality and biological changes in temperate streams and also provide scientific basis for appropriate water quality management practices for stream ecosystems. To protect and sustain the stability of fish abundance and structure in stream ecosystems anthropogenic activities which are responsible for destroying stream habitat, Moreover, to protect these vulnerable species, the local government should strengthen fishery management to counter against illegal fishing.

## Supporting information

S1 Data (XLSX)

S2 Data (XLSX)
