# Expression of Concern: Spatiotemporal variation in fish species distribution and abundance in the Vaishav stream, Kashmir Himalaya–India

**DOI:** 10.1371/journal.pone.0354287

**Published:** 2026-07-22

**Authors:** The PLOS One Editors

The *PLOS One* Editors issue this Expression of Concern for this article [1] because of concerns identified about the peer review process. Readers are advised to interpret the article [1] with caution.
